# Multiannual patterns of genetic structure and mating type ratios highlight the complex bloom dynamics of a marine planktonic diatom

**DOI:** 10.1038/s41598-024-56292-y

**Published:** 2024-03-12

**Authors:** Maria Valeria Ruggiero, Marina Buffoli, Klara K. E. Wolf, Domenico D’Alelio, Viviana Di Tuccio, Ernestina Lombardi, Francesco Manfellotto, Laura Vitale, Francesca Margiotta, Diana Sarno, Uwe John, Maria Immacolata Ferrante, Marina Montresor

**Affiliations:** 1 Department of Integrative Marine Ecology, Stazione Zoologica Anton Dohrn, Naples, Italy; 2 Institut für Marine Ökosystem- und Fischereiwissenschaften, Universität Hamburg, Hamburg, Germany; 3 Limnological Institute, Environmental Genomics, University of Konstanz, Konstanz, Germany; 4 Department of Research Infrastructures for Marine Biological Resources, Stazione Zoologica Anton Dohrn, Naples, Italy; 5 Alfred-Wegener-Institute, Helmholtz Centre for Polar and Marine Research, Bremerhaven, Germany; 6 Helmholtz Institute for Functional Marine Biodiversity, Oldenburg, Germany; 7 Oceanography Section, National Institute of Oceanography and Applied Geophysics, Trieste, Italy

**Keywords:** Ecology, Genetics, Molecular biology, Ecology

## Abstract

Understanding the genetic structure of populations and the processes responsible for its spatial and temporal dynamics is vital for assessing species’ adaptability and survival in changing environments. We investigate the genetic fingerprinting of blooming populations of the marine diatom *Pseudo-nitzschia multistriata* in the Gulf of Naples (Mediterranean Sea) from 2008 to 2020. Strains were genotyped using microsatellite fingerprinting and natural samples were also analysed with Microsatellite Pool-seq Barcoding based on Illumina sequencing of microsatellite loci. Both approaches revealed a clonal expansion event in 2013 and a more stable genetic structure during 2017–2020 compared to previous years. The identification of a mating type (MT) determination gene allowed to assign MT to strains isolated over the years. MTs were generally at equilibrium with two notable exceptions, including the clonal bloom of 2013. The populations exhibited linkage equilibrium in most blooms, indicating that sexual reproduction leads to genetic homogenization. Our findings show that *P. multistriata* blooms exhibit a dynamic genetic and demographic composition over time, most probably determined by deeper-layer cell inocula. Occasional clonal expansions and MT imbalances can potentially affect the persistence and ecological success of planktonic diatoms.

## Introduction

The amount of genetic variation is one of the key factors that influence species’ evolutionary response to environmental changes. Intraspecific genetic diversity can be impacted by shifts in selective pressures due to climatic changes, bottleneck effects, or ecological disturbances altering biological or demographic processes^1,2^. In turn, experimental studies have highlighted the ecological implications of intraspecific diversity on the structure of natural communities^3,4^.

Unicellular eukaryotes, i.e. protists, often characterised by large population size and high division rates, can potentially evolve very fast^5,6^ and represent interesting models to explore mechanisms driving genetic diversification and demographic dynamics. Population genetic studies carried out with microsatellite markers have generally reported high levels of genetic diversity and structure in protist populations^7^, although exceptions occur^8^. Limited gene flow and isolation by distance was detected for the pennate diatom *Pseudo-nitzschia pungens* at a global scale^9^, whereas a global gene flow has been reported for the centric diatom *Thalassiosira rotula*, in which environmental and ecological factors seem to select distinct sympatric populations^10^. Genetic divergence was also observed at relatively limited spatial scales as shown by the distinct populations of the diatom *Skeletonema marinoi* across a salinity gradient from the North Sea to the Baltic Sea^11^. Few studies have addressed the genetic structure of protists over a temporal scale, i.e. between blooms in subsequent years and changes in the genetic fingerprinting were at times detected^8,12–14^.

The genetic make-up of populations is also influenced by specific life cycle traits, which are still poorly investigated in protists^15–17^. These organisms duplicate by mitotic divisions and the magnitude and temporal extension of blooms can potentially impact the genetic structure of populations via the accumulation of mitotic mutations^6^. Moreover, many species bloom only in a restricted period of the year and bottlenecks between subsequent blooms can be responsible for genetic drift and founder effects^14^. There is also evidence for sexual reproduction in an increasing number of unicellular organisms^18^ and the frequency of sexual recombination can be a major driver of genetic variation^19^.

The marine diatom *Pseudo-nitzschia multistriata* is regularly recorded in the Gulf of Naples (GoN; Tyrrhenian Sea, Mediterranean Sea) since 1995^20^. This species was selected as a model for genetic and genomic studies^21^ because it has a distinctive sigmoid cell shape that allows clear identification in light microscopy, it does not present cryptic diversity^14^ and produces the neurotoxin domoic acid responsible for Amnesic Shellfish Poisoning^22,23^. As in the majority of diatoms, the average cell size of a population gradually decreases following mitotic divisions and large sized initial cells are only produced during the sexual phase^24^. *P. multistriata* has a heterothallic mating system, i.e., the population is constituted of cells of opposite mating type (MT + and MT−) that need to get in contact to start the sexual phase^25^. The sex-determining gene has been recently identified and the analysis of allelic pattern in the upstream portion of this gene allows the assignment of MT to isolated strains^26^.

Blooms are key events in diatom life histories, and are generally characterised by high genotypic diversity; at the same time, they should be ideal settings for sexual reproduction to occur, facilitating encounter rates between opposite MTs. We here illustrate the genetic structure and temporal connectivity of *P. multistriata* populations at the Long Term Ecological Research site MareChiara (LTER-MC^27^) in the GoN during several blooming seasons. We pooled new and published data obtained with classical microsatellite fingerprinting over the period 2008–2020, and applied Microsatellite Pool-seq Barcoding (MPB), which allows to analyse microsatellite alleles in natural populations independently from strain isolation^13^. We integrated these data with the assessment of two key demographic parameters: estimates of sexual recombination and mating type ratios in natural populations to gain insights on their impact on the genetic make-up in different blooms.

## Results

### Pseudo-nitzschia multistriata in the Gulf of Naples

**Figure 1:**
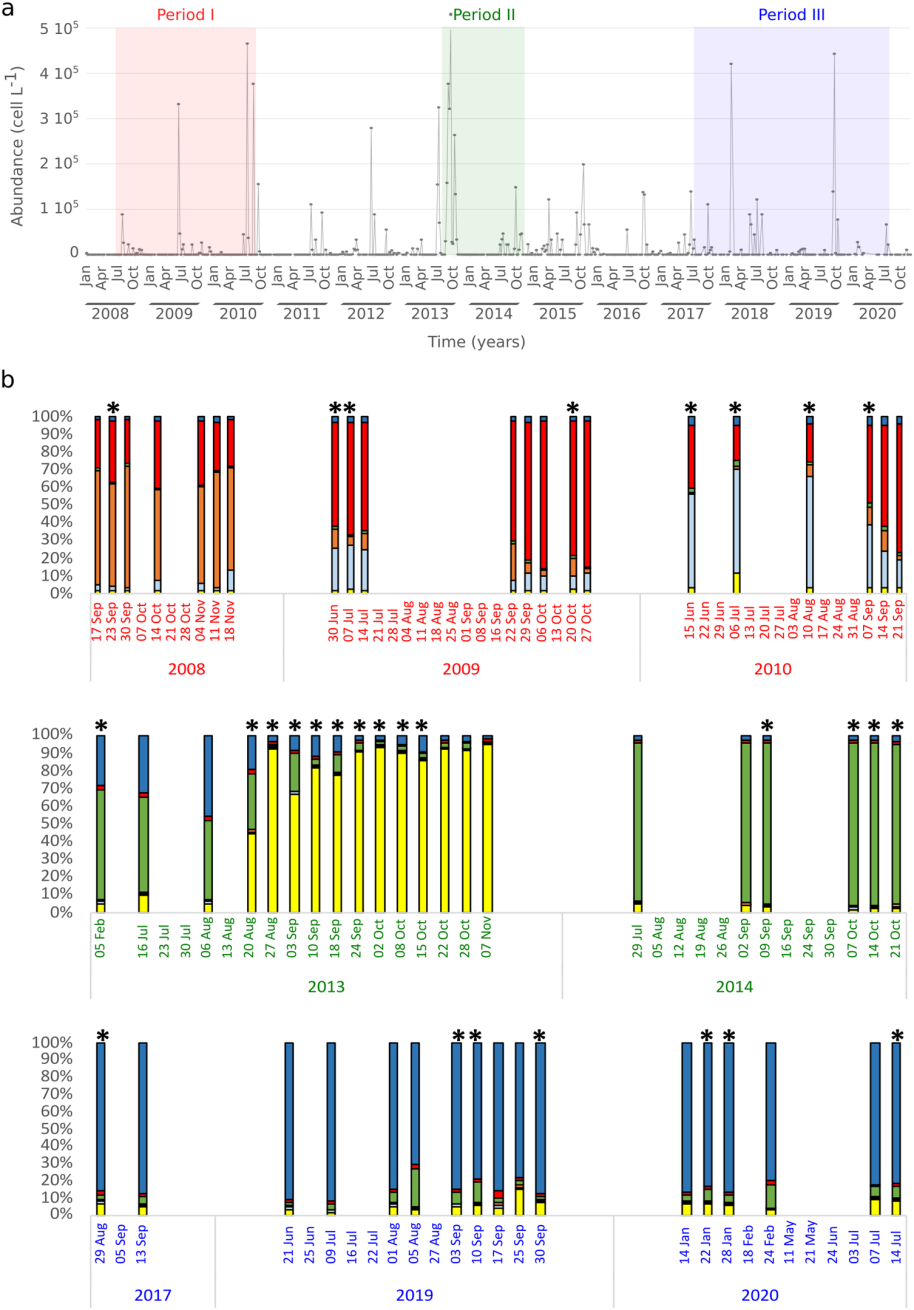
*Pseudo-nitzschia multistriata* cell abundances and population genetic structure. (**a**) Cell abundances (cells L^−1^) recorded at the LTER-MC in the Gulf of Naples from 2008 to 2020; the periods in which genetic analyses were carried out are marked with different colours. (**b**) For each of the studied periods, percentage composition of the genetic clusters based on microsatellite fingerprinting and obtained with STRUCTURE (K = 6) are represented; asterisks mark sampling dates in which *P. multistriata* cell concentration was ≥ 10^4^ cells L^−1^.

*Pseudo-nitzschia multistriata* was first recorded at the LTER-MC site in the GoN in 1995 (Supplementary Fig. 1). In the first years of occurrence (1995–2004), blooms of *P. multistriata* were recorded at the end of summer/beginning of autumn with scattered low abundances between January and March^20^, but its phenology changed in the subsequent years. Since 2005 and in the years covered by this study (2008–2020), the species was also detected in summer—June and July—when the highest annual concentration values often occurred (Fig. 1a). Cell abundances in winter were generally lower, but high values were recorded in January 2018 (4.2 × 10^5^ cells L^−1^) and January 2020 (2.77 × 10^4^ and 1.71 × 10^4^ cells L^−1^). *P. multistriata* was recorded over a broad range of temperatures spanning from 14 °C during the winter to values > 26 °C in the summer months (Supplementary Fig. 2a), although records of the species have been always more frequent during the warmer periods of the year. Salinity in surface waters at LTER-MC is generally between 37 and 38, with lower values linked to high rain events, and the species was recorded over a broad range of values (Supplementary Fig. 2b). No relationships could be found with concentration of inorganic nutrients, as shown by the non significant regression values (Supplementary Fig. 2c, d, e).

### Population genetic structure with microsatellite fingerprinting

In order to obtain a comprehensive picture of the genetic structure of the population over a long time interval, we integrated the microsatellite dataset produced in this study (2017, 2019–2020) with the data produced in two previous studies^8,28^, which covered the time intervals 2008–2010 and 2013–2014, respectively (Fig. 1a); see Supplementary Table 1 for a summary of the data considered in the different years. The whole dataset included 1,133 genotyped strains isolated over 59 sampling dates from 2008 to 2020 (Supplementary Table 2; Supplementary Data). An average number of 15 alleles (from 7 to 30) was recorded in the five microsatellite loci.

**Figure 2:**
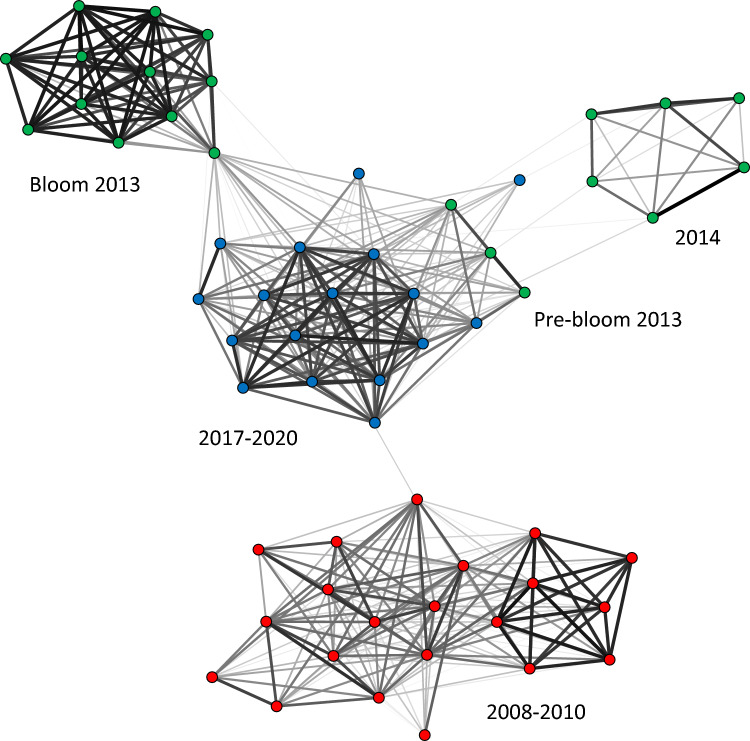
Network analysis of microsatellite fingerprinting data. The Network is based on the F_ST_ pairwise matrix; nodes represent sampling dates; node colours are coded according to the three periods with different genetic structure identified by STRUCTURE (see Fig. 1b); width of the edges connecting nodes is proportional to genetic similarity.

The results of STRUCTURE analysis showed the presence of six distinct genetic clusters (Fig. 1b). Three of them were almost exclusively recorded in the period 2008–2010, with samples from the first year distinct from those of the other two. A sharp shift introduced the second period (2013–2014) where the samples collected before the late summer/autumn bloom in 2013 had a genetic profile more similar to the 2014 population. The 2013 bloom had a distinct genetic profile and was remarkable in being characterised by the dominance of a single genotype^8^ (Supplementary Table 2). The sixth cluster became dominant from 2017 onward. The Network analysis built with F_ST_ data provided a similar temporal pattern, identifying one cluster with samples from 2008 to 2010, one cluster including samples of the clonal bloom in 2013 (from August 20 onwards) distinct from the pre-bloom samples, a cluster with the 2014 samples and the cluster grouping samples collected between 2017 and 2020 (Fig. 2).

A total of 523 multilocus genotypes (MLGs) were sampled between 2008 and 2020; however, the number of genotypes retrieved in more than one bloom was extremely low (Supplementary Fig. 3). The vast majority of genotypes were confined to a specific year and were never sampled again in subsequent blooms. The clustering pattern reflected the results of STRUCTURE analysis. Notably, no genotypes from the period 2008–2010 were recorded in the subsequent years; the genotype responsible for the late summer bloom of 2013 was never recorded again and only five genotypes from the pre-bloom period in 2013 (CCCC, AA, MMMM, O and OOOO) were recorded in 2019 and two in 2020 (AA and WWWW; Supplementary Fig. 3).

**Figure 3:**
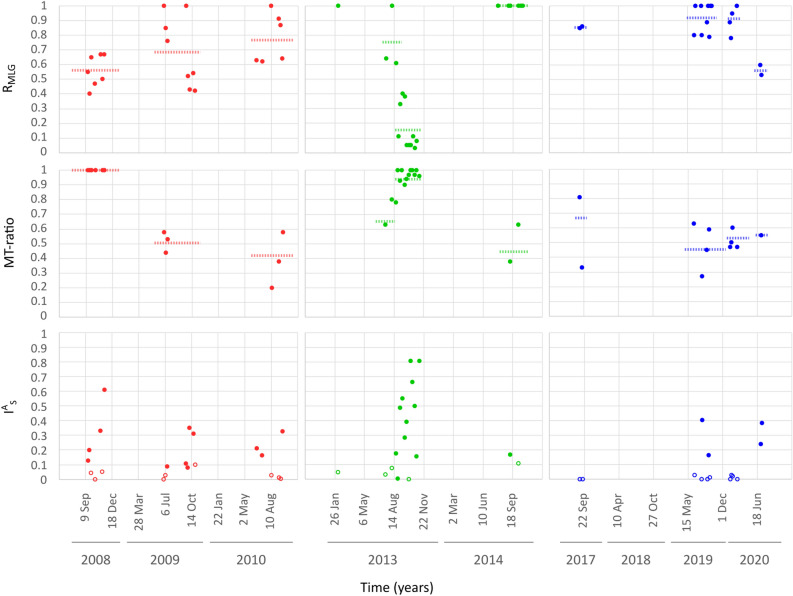
Genotypic richness, mating type ratio and association index. Values calculated for the individual sampling dates are illustrated. For genotypic richness (R_MLG_, upper panel) and mating type ratio (MT-ratio, middle panel) average values for the pre-bloom phase and the clonal bloom in 2013, and for the winter and summer blooms in 2020 are also represented by dotted horizontal lines. For the index of association (I_A_^S^, bottom panel), empty symbols represent non-significant values of I_A_^S^, i.e., linkage equilibrium; full symbols represent significant values of I_A_^S^, i.e., linkage disequilibrium.

Genotypic richness (R_MGL_) was relatively low in 2008, spanning between 0.40 and 0.67 (average value 0.56) and it increased in 2009 (average 0.69) and 2010 (average 0.78) (Fig. 3, upper panel; Supplementary Table 2). In the period preceding the clonal bloom of 2013 (from July 16 to August 20), R_MGL_ values spanned between 1.0 and 0.61 and dramatically dropped afterwards, with extremely low values until the end of the bloom. Higher values of genotypic richness were recorded in the subsequent years, with a decrease in the summer bloom of 2020 (average: 0.56).

### Mating type ratios and evidence for recombination

*Pseudo-nitzschia multistriata* has a heterothallic mating system and the success of sexual reproduction depends on the contemporary presence of the two complementary mating types (MTs). We determined mating type, via crossing or genetic analysis, for 969 strains isolated from natural samples from different years (Fig. 3, middle panel; Supplementary Table 2). The MT ratio (as % of MT + strains) was closer to equilibrium in most of the years, with the exception of 2008 and 2013. All the 67 tested strains in 2008 turned out to be MT + as did the vast majority of the 485 strains isolated during the clonal bloom (from August 20 onwards; avg MT ratio = 0.95; std = 0.06).

Unbalanced MT ratio could decrease the occurrence of sexual reproduction; we thus calculated the index of association (I^A^_S_) as a proxy for the occurrence of sexual reproduction. The index of association provides an assessment of the linkage among microsatellite loci, which is expected to be high (significantly high values of I^A^_S_ = Linkage Disequilibrium, LD) when clonal growth prevails and low (non-significant values of I^A^_S_ = Linkage Equilibrium) when alleles recombine freely during sexual reproduction. LD was detected in 2008, 2009, and during the clonal bloom of 2013 (Fig. 3, lower panel; Supplementary Table 2). The samples preceding the 2013 bloom and the majority of samples from the years 2010, 2014, 2017, 2019 and 2020—with the exception of the summer bloom—were at equilibrium, suggesting that genetic recombination was occurring in those populations. Notably, a highly significant negative correlation (Pearson’s R = -0.91) was observed between genotypic diversity (R_MLG_) and I^A^_S_, indicating that the amount of LD in populations is mainly affected by clonal replication of genotypes.

### Microsatellite Pool-seq barcoding (MPB)

**Figure 4:**
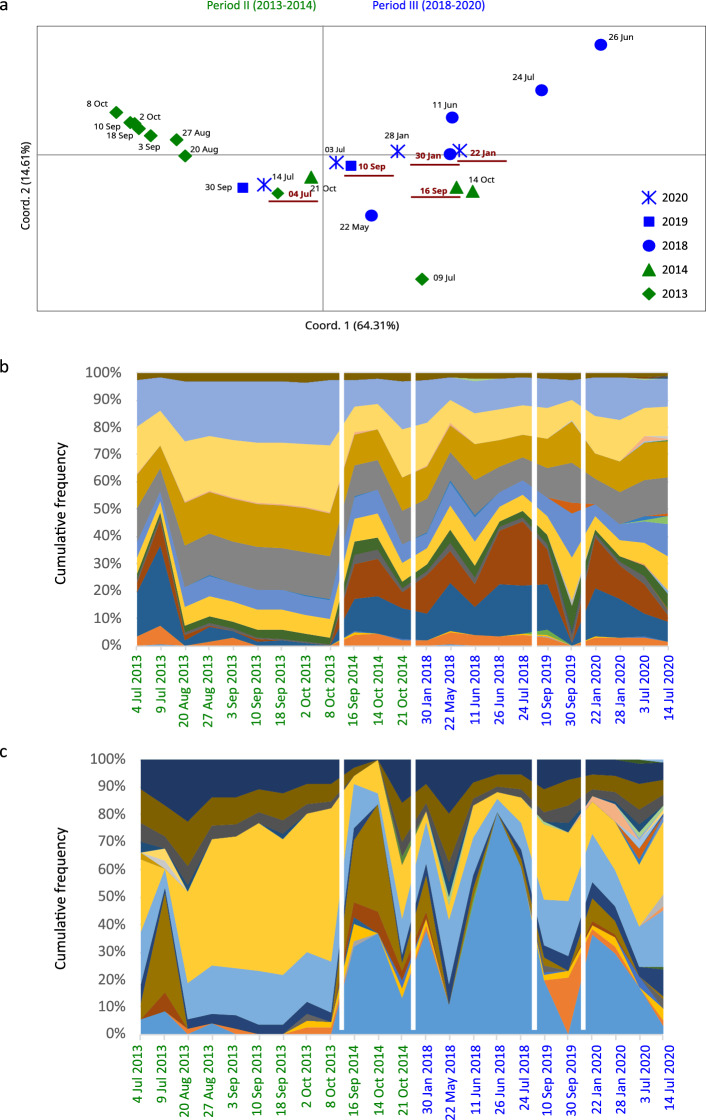
Microsatellite Pool-seq Barcoding of natural samples. (**a**) PCoA built with F_ST_ pairwise matrix. Samples collected in each year are represented by different symbols; the date of first sample of the year is marked in red. (**b**, **c**) Allelic frequencies, in percentage, at the two loci, PNm1 (**b**) and PNm6420 (**c**).

The analysis of microsatellite data with fingerprinting is based on individually isolated strains; although numbers can be in the order of hundreds, they represent a subsample of the much larger natural populations. We therefore analysed 23 environmental samples collected during the blooming seasons of 2013–2014 and 2018–2020 (Supplementary Tables 1 and 3) also with MPB, which is based on the PCR amplification and Illumina sequencing of selected microsatellite markers on the entire sampled environmental DNA. The MPB dataset included 3.6 × 10^6^ amplicons for two microsatellite loci, with the number of alleles ranging from 17 (14.10.2014) to 43 (14.07.2020) over the two loci (Supplementary Table 3). The PCoA built on F_ST_ pairwise matrix explained 88% of the variance (Fig. 4a). All samples of years 2014, 2019, 2020, the pre-bloom samples of 2013 and those of the winter bloom of 2018 clustered around the central portion of the bi-dimensional plot, while the samples from the summer blooms of the two latter years followed distinct trajectories indicating their different genotypic/allelic makeup. It is worth noting that the first samples of each year were located at relatively limited reciprocal distance in the central portion of the plot (Fig. 4a), thus suggesting that they share a similar allelic profile.

MPB allowed a finer level of analysis of the alleles of the two microsatellite loci (Fig. 4b, c). All environmental samples of the clonal bloom of 2013 (from 20.08.2013 to 08.10.2013, for a total of 7 samples) showed an almost identical ratio of allelic composition in the two loci, thus providing a further support for the clonality of the bloom detected with the fingerprinting approach applied to the isolated strains. Also the samples from the summer bloom in 2018 took a distinct trajectory but, unfortunately, we do not have fingerprinting data of this year for comparison.

The MPB approach also allowed to discriminate between homoplastic –same amplicon size but differing in the sequence of the target region and/or of the flanking region—and heteroplastic alleles, which differ in the sequence length i.e., in the number of copies of the core repeat (Supplementary Table 3). In the heteroplastic alleles of locus PNm1, SNPs were not randomly distributed in the flanking regions. Four different types of sequence stretches were detected at the 5’ and four types at the 3’ flanking regions. Most of the alleles presented the same type of flanking regions, while a recombined pattern was observed for eleven alleles (Supplementary Table 4; Supplementary Fig. 4), with frequencies ranging from 0.3 to 5.5%. Recombinant alleles were recorded only in 2019 and 2020. The 3’ flanking sequence of locus PNm6420 was too short to allow any analysis for this locus.

## Discussion

### Population genetic structure through time

The analysis of 1133 strains genotyped with microsatellite markers, collected over eight years in the period 2008–2020, allowed a comprehensive assessment of the overall temporal pattern of connectivity of *P. multistriata* populations in the GoN. A major discontinuity separated the populations of the first three years (2008–2010) from those of the subsequent period. This was clearly shown by the STRUCTURE and Network analyses and supported by the fact that none of the individual genotypes of this period were recorded in subsequent years. The populations of the period 2013–2020 showed a higher level of genetic connectivity, with the exception of the clonal expansion event in 2013. The MPB results of natural samples confirmed the “clonality” of this long lasting bloom in which both microsatellite markers showed an almost identical allelic ratio. We have to consider, however, that there were gaps in our dataset since genetic analyses were not carried out every year. As an example, the lack of genotyped strains in 2011 and 2012 does not allow to test in which year, between 2010 and 2013, the change of the genetic profile occurred, and if it was a sudden change or a gradual transition; the same holds for the changes detected between 2014 and 2017. Nevertheless, our comprehensive dataset constitutes, to the best of our knowledge, the longest time series of population genetic data for marine diatoms and clearly shows that the genetic profile of *P. multistriata* changes over time with different dynamics among years.

Results of the STRUCTURE analysis show that the pattern of genetic clusters remains almost constant within the annual blooms, with the exception of the clonal expansion event detected in summer-autumn 2013. Individual blooms were genotyped at multiple sampling dates and the stable genetic profile supports the fact that we were following the same population in the GoN.

Differences in the genetic structure of the *P. multistriata* population over the years bring up the question on the origin of the distinct annual blooms and their temporal development. *Pseudo-nitzschia* species do not produce benthic resting stages^29,30^ whose germination can constitute an inoculum for genetically distinct blooms, as hypothesised for dinoflagellate species producing resting cysts^12,31^. An insight comes from the results of the MPB data, which show that samples collected at the beginning of each annual bloom were located at relatively short distance in the central portion of the PCoA plot, from where the subsequent samples take their own trajectory in the temporal space. This suggests that the inoculum of the blooms derives from a common reservoir, most likely the diluted population in the deeper water column. Indeed, blooms of *P. multistriata* and other *Pseudo-nitzschia* species in the GoN are confined to the surface layer but cells remain in the deeper layers at extremely low concentrations all over the annual cycle^32^. We thus postulate that *Pseudo-nitzschia* blooms likely originate from periodic inocula from the deeper reservoir, which increase growth in the surface layer and can genetically evolve at the microscale of their growth season. Remarkable examples are the distinct trajectories in the MPB plot of the clonal bloom in summer-autumn 2013 and the summer bloom of 2018.

In line with the transition in the population genetic profile, a regime shift in the physical structure of the water column was detected between the first two decades of this century, with a remarkable shallowing of the mixed layer depth (MLD) coupled with a lengthening of the period in which the water column was stratified^33^. This might have favoured the maintenance of a genetically more homogeneous population in the water column after 2010, which is mirrored by the higher connectivity between blooms in the period 2017–2020.

### Genetic recombination and mating type ratios

To counteract the progressive cell size reduction that occurs during mitotic divisions, the vast majority of diatoms produce large sized cells within the sexual reproduction phase. However, records of sexual events in marine natural populations are extremely scanty and, to the best of our knowledge, reported only in blooms of *Pseudo-nitzschia* species^34,35^ and of *Fragilariopsis kerguelensis*^36^. These rare findings can be related to the ephemeral nature of sexual events, which last only a few days, and/or to the low abundance of sexual stages, which can easily be overlooked by the counting methods in use. An attempt to infer the frequency of sexual events for *P. multistriata* in the GoN was based on the long term monitoring the cell size of natural population, considering the appearance of large-sized populations as a proxy for sexual reproduction^20^. That study demonstrated a regular biennial occurrence of sex; however, this regularity was apparently weaker in 2008–2010 in concomitance with the expansion of the blooming season of this species^28^. MPB allowed also to identify rare recombinant alleles, suggesting that this method could provide additional indication on the occurrence of sexual reproduction in natural populations.

Linkage equilibrium, a proxy for the occurrence of sexual reproduction, was observed in 2010, 2014, 2017 and 2019, indicating a significant contribution of recombination events in shaping the genetic profile of the *P. multistriata* population. Linkage disequilibrium (LD) was instead detected in periods characterised by low genotypic richness, confirming that the amount of LD in the population is mainly affected by clonal replication of selected genotypes. Overall, our results suggest that genetic recombination, i.e., sexual reproduction, has a homogenising effect on the genetic pool of the population, counteracting the build-up of genetic structure that can arise during some blooms.

In organisms with two mating types—as *P. multistriata*—sexual reproduction requires the contemporary presence of two MT, whose ratio at equilibrium is expected as 50:50^37^. A marked deviation from these values may represent a demographic constrain for the occurrence of sexual reproduction. MT ratio was indeed roughly at equilibrium in the majority of samples, but two major exceptions occurred in 2008 and 2013, when a marked dominance of MT + strains was detected for the whole duration of the blooms. Within the summer bloom in 2013, the dominance of MT + was linked with extremely low genotypic diversity, demonstrating that the population was largely dominated by a clonal MT + genotype^8^. However, the situation was different in 2008 when the dominance of MT + genotypes was not associated with particularly low genotypic diversity. While the first case can be interpreted as an epidemic expansion of a particularly fit genotype, as reported for cyanobacteria or parasites^38,39^, the dominance of genetically different MT + strains in 2008 is puzzling. A MT-specific mortality for MT- strains can be hypothesised, but this requires further investigations.

Within a study aimed at testing reproductive barriers among cryptic *Pseudo-nitzschia* species, sexual reproduction was never obtained in > 700 crosses of 25 strains of *P. delicatissima *sensu stricto (identified as *P. delicatissima2* in^40^) isolated at LTER-MC on several occasions in 2004^40^. One explanation could be that all isolated strains belonged to the same mating type, thus suggesting that unbalanced MT ratio can occur also in other species of this genus. The fact that blooms in some years were apparently constituted by only one mating type did apparently not impact the population of the following year, in which a balanced MT ratio was recorded. This finding further supports the hypothesis that the demographic and genetic profile of *P. multistriata* blooms strongly depends on the inoculum populations. A tighter monitoring of the early bloom phases coupled with the genetic and MT characterisation of the deeper populations may shed light on the mechanisms driving bloom development of heterothallic diatoms.

### Conclusion

We have shown that biological features, as mating types and frequency of sex, can play a considerable role in shaping the structure of *P. multistriata* populations; blooms can be remarkably different from year to year, both in terms of genotypic diversity, temporal genetic connectivity, occurrence of genetic recombination and ratio of mating types. The genetic and demographic profile of the inoculum population should play an important role for the blooming phase and we hypothesise that blooms are started by advection of cells from the deeper layers of the water column. Remarkably, in rare occasions, blooms can be dominated by a clonal population as observed during summer/autumn 2013^8^; this result was here confirmed by MPB sequencing of the whole natural population. The eco-evolutionary drivers of this exceptional event remain to be elucidated and it would be interesting to know how widespread clonal expansion events are among blooming diatoms and to identify the genetic basis of the selected most competitive clonal lineages. Another aspect that deserves consideration is the mechanism leading to mating type unbalance, which can potentially have profound impacts on the persistence and diversity of heterothallic diatom populations. Sexual reproduction is required by *P. multistriata* to produce large cells and counteract the progressive miniaturization of cell size that follows vegetative cell division; if populations are constituted by only one mating type, sex cannot occur. The potential of the MPB approach to follow over time the genetic structure of natural populations, and the availability of genomic resources for this model diatom^21^, will hopefully allow to further address the questions that emerged from this study and understand the mechanisms that drive intraspecific genetic diversity at the micro-evolutionary scale.

## Methods

### Sample collection and analysis

Seawater samples were collected weekly with Niskin bottles at 0.5 m at the Long-Term Environmental Research site MareChiara (LTER-MC; 40°48.5′ N, 14°15° E; Supplementary Fig. 5) located about two nautical miles offshore in the GoN (Tyrrhenian Sea, Mediterranean Sea).

Samples for cell enumeration were fixed with neutralised Lugol’s solution. *Pseudo-nitzschia multistriata* cells were identified and enumerated using an inverted light microscope (Zeiss Axiovert, Carl Zeiss, Oberkochen, Germany) at 400×magnification, following the Utermöhl method^41^.

In order to obtain material to establish monoclonal cultures, a phytoplankton sample was collected with a 20 µm mesh-sized phytoplankton net towed in surface waters for a few minutes. In the lab, the sample was diluted with filtered seawater and dispensed in 24-well culture plates. Individual cells or short chains were isolated with a micropipette; only one cell was isolated from each culture well to avoid the possibility of isolating two recently divided sister cells. Cultures were grown in f/2 culture medium^42^ at a temperature of 18 °C, an irradiance of 50 μmol photons m^−2^ s^−1^ and a photoperiod of 12:12 h L:D. Monoclonal cultures established throughout the study period were utilized for both microsatellite fragment analysis and mating type determination (see below).

For Microsatellite Pool-seq Barcoding (MPB), ~ 3 L of seawater from the Niskin bottle closed at 0.5 m were filtered on cellulose ester filters (47 mm diameter, 1.2 μm pore-size, EMD Millipore, USA). Filters were immediately frozen with liquid nitrogen and stored at − 80 °C.

Temperature and conductivity were acquired with a SBE 19plus (Sea-Bird Scientific, U.S.) multi-parametric probe. Samples for dissolved inorganic nutrient analyses (NO_3_, PO_4_ and SiO_4_) were collected in 20 mL high-density polyethylene vials and immediately stored at − 20 °C. Analyses were carried out with a five-channel continuous flow autoanalyzer (Flow-Sys, Systea, Italy), according to Hansen and Grasshoff (1983)^43^.

### Microsatellite fragment analysis

Genotype matrices from five microsatellite loci (PNm1, PNm2, PNm3, PNm7 and PNm16) were retrieved from Tesson et al. (1914)^28^ for years 2008–2010 and from Ruggiero et al. (2018)^8^ for years 2013–2014. For years 2017, 2019 and 2020, genotype data were collected as described below.

For DNA extraction, cultures were harvested by centrifugation and then processed as in Tesson et al. (2011)^44^. Microsatellite genotyping was performed as in Ruggiero et al. (2018)^8^; microsatellite fragment data are available in Supplementary data. The average number of alleles per locus, the expected unbiased heterozygosity^45^, were calculated with Genalex v.6.5^46^. Genotypic richness per each sampling date was calculated as: R_MLG_ = (MLG−1)/(N−1), where MLG is the number of Multi-Locus Genotypes and N is the total number of strains in the sample. The independence of alleles assortment was tested with the software LIAN^47^, computing the number of loci at which two MLGs differ and then comparing the observed variance of the mismatch values (Vd) with respect to the variance expected under linkage equilibrium (Ve). The association index I^S^_A_ was calculated as: I^S^_A_ = [1/(n − 1)]⋅[(Vd/Ve) − 1]. The null hypothesis of linkage equilibrium was tested via Monte Carlo simulations (1000 iterations).

The number of genetically distinct clusters and their occurrence through time were inferred using a Bayesian cluster analysis performed with STRUCTURE. The most probable number of clusters (K; populations) was estimated following the Evanno method^48^, using the program STRUCTURE HARVESTER (https://taylor0.biology.ucla.edu/structureHarvester/). Population structure was obtained applying a burn-in of 25,000 iterations, with 75,000 MCMC repetitions from K1 to K20. The parameters used to infer genetic structure were an ancestry model with admixture, along with a correlated frequency model. The LOCPRIOR assumption was not applied. The Assignment Probability (AP) threshold for a sample to be assigned to a population was set at 90%.

A network analysis was conducted on fragment data based on the F_ST_ pairwise matrix using the software EDENnetwork^49^.

### Mating type determination

Mating type was determined by crosses of a selection of strains isolated in the different years and/or by genotyping the region upstream of the gene MRP3^26^. Crosses were carried out in 6-well culture plates (Corning Costar) filled with 3 mL of f/2 medium. Each one of the tested strains was inoculated with two reference strains of known mating type (MT + and MT-) in separate wells at a final cell concentration of ~ 6 × 10^3^ cells mL^-1^ and incubated at the culture conditions indicated above. Incubations were inspected using a Zeiss Axiovert light microscope for five consecutive days to detect the presence of sexual stages (gametes, auxospores, and initial cells), evidence for successful sexual reproduction in crosses with complementary MT^25^.

In *P. multistriata* the mating type can be assigned by amplifying a multiallelic region upstream of the gene MRP3^26^. The alleles are identified by their different size and, in the population under study, the presence of a specific allele (allele A) has been shown to be responsible for MT + determination. To assign the mating type, PCRs were performed using Q5® High-Fidelity DNA Polymerase (New England Biolabs) according to the manufacturer’s instructions using the F147prom/R147p10 and F147p3/R147p3 primer pairs located upstream of the gene MRP3^26,50^. Primer sequences used for PCR analysis are reported in Russo et al. (2018, 2021)^26,50^. A subset of samples was also analysed via fragment analysis. The forward primer F147prom was 5’-labelled with the fluorophore VIC (Life Technologies, Carlsbad, CA, USA) and PCR was performed using the Type-it Microsatellite PCR Kit (Qiagen Ltd., Venlo, The Netherlands) and analysed on an Automated Capillary Electrophoresis Sequencer 3730 DNA Analyzer (Life Technologies). Size of fragments was determined using the software PEAK SCANNER v1.0 (Applied Biosystems, Foster City, CA, USA).

### Microsatellite Pool-seq barcoding (MPB)

DNA was extracted, amplified, and processed as in Wolf et al. (2021)^13^^.^ The procedure was followed with minor changes related to the properties of the microsatellites employed, as described in detail in Supplementary Methods. Two microsatellite loci, PNm1 and PNm6420^8^, were amplified using Illumina primers. In brief, PCR products were visualised on an agarose gel, bands of the target size were excised and purified using NucleoSpin Gel and PCR Clean-up Kit (Macherey–Nagel, Düren, Germany). In a second PCR reaction dual indices and Illumina sequencing adapters were attached using the Nextera XT Index Kit. The final PCR products were purified using NGS clean-up mag-beads (Beckman Coulter, Brea, CA, USA), validated using LabChip GX Touch Nucleic Acid Analyzer (Agilent Technologies, Santa Clara, CA, USA) and pooled at equimolar ratios. MPB libraries were sequenced using the MiSeq System with the v3 2 × 300 bp Paired-end Kit (Illumina). To test the quantitative representation of the contained alleles (Validation1), the same PCR reaction was carried out with artificial mixtures of DNA obtained by pooling DNA extracted from five strains at different proportions.

### MPB: Bioinformatics and data processing

Raw sequences produced by Illumina MiSeq were converted into amplicon contingency tables. Demultiplexing, trimming, merging forward and reverse sequences, truncating and filtering procedures were carried out following Wolf et al. (2021)^13^. About 4 million amplicons for primer set PNm1 and 2 million for primer set PNm6420 were obtained. Amplicon tables for the two microsatellite loci were further processed using R software (version 4.0.4 (2021-02-15). Singletons (0.13% of PNm1 and 0.77% of PNm6420 amplicons), were discarded. A repeat sequence filter specific for each locus was applied and alleles with less than 3 repeat units were excluded. These steps removed 0.001% of the reads for PNm1 and 8.4% for PNm6420. All MBP samples contained a minimum of 1000 amplicons.

Sample libraries were normalised to the median of total amplicon numbers in all samples and allele frequencies were expressed as percentages of the total amplicons per sample. Homoplastic alleles, with the same length but different sequence, were assigned unique names (e.g., 114.1 and 114.2). A locus-specific allele abundance filter was applied to find the best balance between removal of erroneous alleles and retaining rare alleles. Based on the number of heteroplastic alleles found by fragment analysis, we excluded alleles with a frequency < 0.65% for PNm1, and < 1.65% for PNm6420.

A 2-step validation was performed to compare results of MPB and conventional microsatellite analyses in terms of identification and quantification of the allelic composition of *P. multistriata* (see Supplementary Methods).

A F_ST_ pairwise matrix per sampling date was obtained from allelic frequencies using the software POPTREEW^51^. A principal component analysis (PCA) based on the F_ST_ matrix was built in Genalex V.6.5. The analyses included both homoplastic and heteroplastic alleles. Replicates represented by environmental samples collected at the same sampling date were merged by calculating the average of the allele frequencies for each allele.

### Supplementary Information

Supplementary Information.

## Data Availability

Data generated during this study are included in this published article and its Supplementary Information files; The Microsatellite Pool-seq Barcoding data are available in the SRA—NCBI repository: https://dataview.ncbi.nlm.nih.gov/object/PRJNA986312?reviewer=ilgcoufnuvbmfokluo212rp0a3.
