# Supplementary material for: Multiannual patterns of genetic structure and mating type ratios highlight the complex bloom dynamics of a marine planktonic diatom
Source: Sci Rep. 2024 Mar 12;14:6028. doi: 10.1038/s41598-024-56292-y (PMC10933277; doi:10.1038/s41598-024-56292-y)
Supplement: Supplementary file 1 — Supplementary Information. [file 41598_2024_56292_MOESM1_ESM.pdf]

SUPPLEMENTARY INFORMATION

Title

Multiannual patterns of genetic structure and mating type ratios highlight the complex bloom dynamics of a marine planktonic diatom

Authors

Maria Valeria Ruggiero[1], Marina Buffoli[1], Klara K.E. Wolf[2], Domenico D'Alelio[1], Viviana Di Tuccio[1], Ernestina Lombardi[1], Francesco Manfellotto[1], Laura Vitale[1,6], Francesca Margiotta[3], Diana Sarno[3], Uwe John[4,5], Maria Immacolata Ferrante[1,6], Marina Montresor[1]*

This document includes:

- **Supplementary Figure 1**: Log transformed weekly abundance ($\log_{10}$ cells L^{-1}) plot of *P. multistriata* at LTER-MC over the period 1995–2020.
- **Supplementary Figure 2**: Scatter plots and relative linear regressions of cell concentration of *P. multistriata vs* environmental parameters measured at LTER-MC at the sampling dates in which the species was recorded in the years 2008-2020
- **Supplementary Figure 3**: Heat map illustrating the number of genotypes that were resampled in different years.
- **Supplementary Figure 4**: Frequency, in percentage, of the recombinant alleles of locus PNm1.
- **Supplementary Figure 5**: Location of the Long Term Ecological Research site MareChiara (LTER-MC) in the Gulf of Naples Tyrrhenian Sea, Mediterranean Sea.
- **Supplementary Table 1**: Summary of the *Pseudo-nitzschia multistriata* datasets presented in this study.
- **Supplementary Table 2**: Summary statistics for the samples of *P. multistriata* collected in 2008–2020 and genotyped by fragment analysis.
- **Supplementary Table 3**: Summary statistics for the environmental samples collected in 2013–2020 and genotyped via Microsatellite Pool-seq Baracoding (MPB).

- **Supplementary Table 4**: List of PNm1 alleles and their composition.
- **Supplementary Methods** for Microsatellite Pool-seq Barcoding (MPB).

SUPPLEMENTARY FIGURES

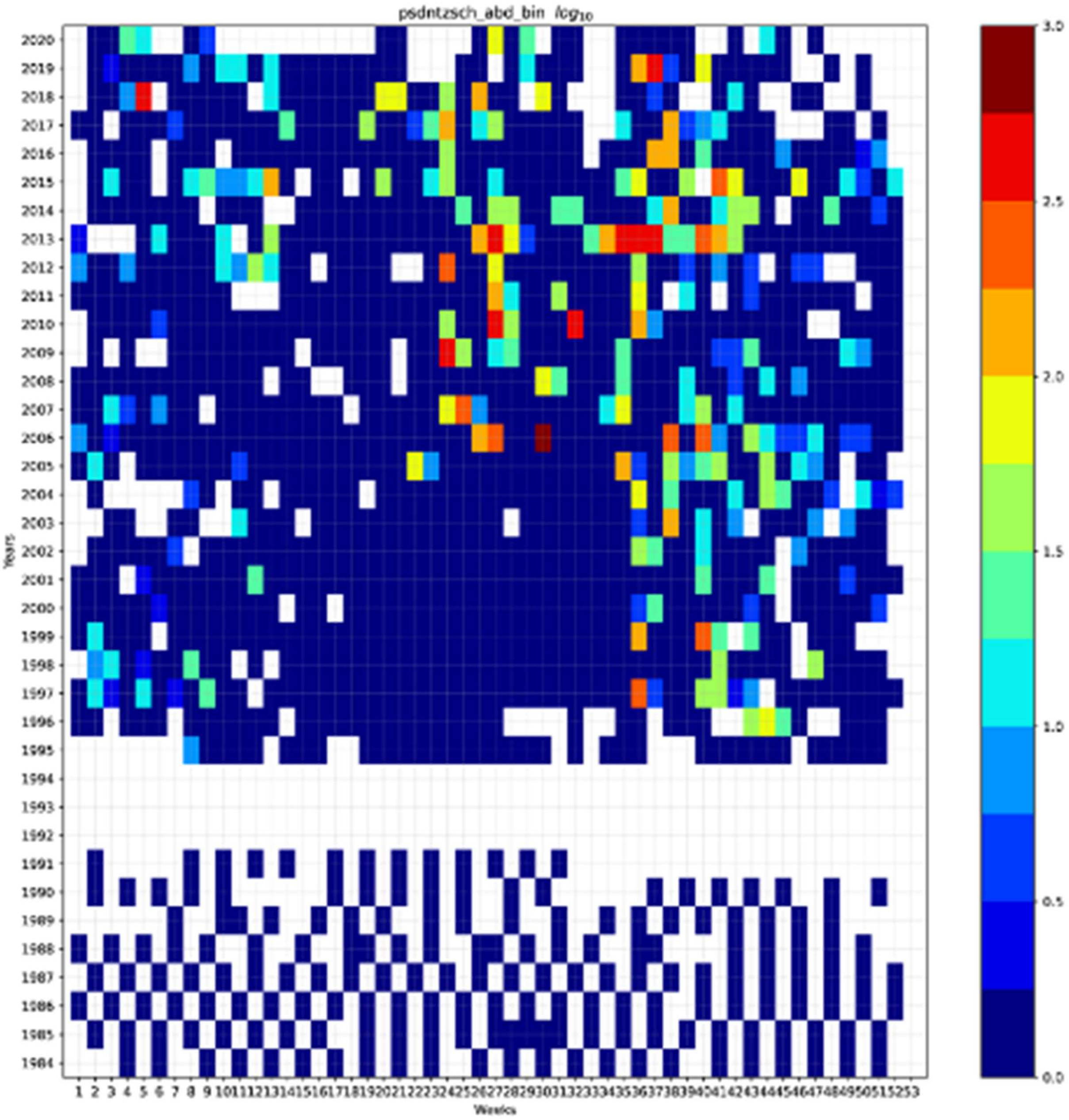

Supplementary Figure 1. Log transformed weekly abundance (log 10 cells L^{-1}) plot of *P. multistriata* at LTER-MC over the period 1984–2020. White squares indicate no sampling. The first sample in which *P. multistriata* was detected was the one collected on week 8 in 1995,

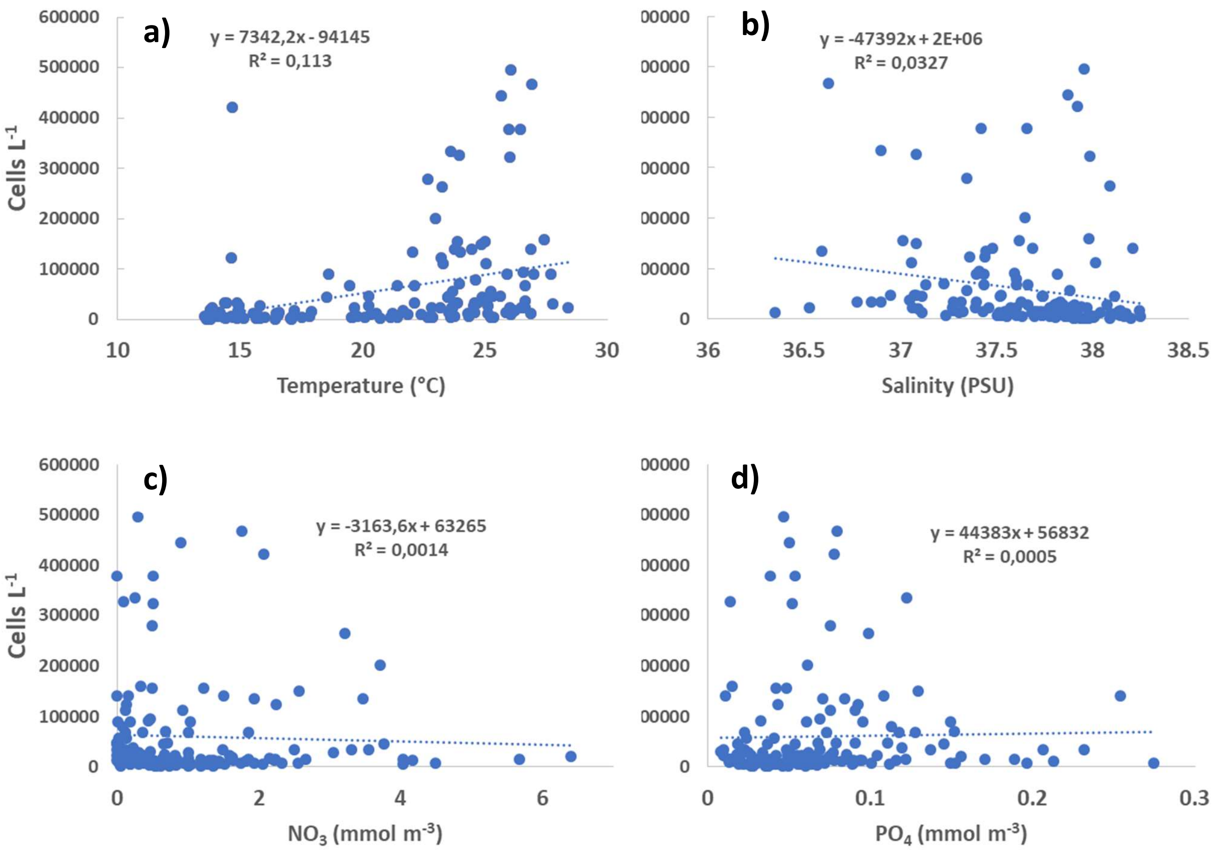

e) Correlation matrix (Spearman):

Variables	PN (cell l-1)	TEMP	PSAL	NTRA	PHOS
PN (cell l-1)	**1**	**0.499**	**-0.415**	-0.168	0.186
TEMP	**0.499**	**1**	-0.195	**-0.541**	-0.097
PSAL	**-0.415**	-0.195	**1**	-0.061	**-0.370**
NTRA	-0.168	**-0.541**	-0.061	**1**	**0.385**
PHOS	0.186	-0.097	**-0.370**	**0.385**	**1**

Values in bold are different from 0 with a significance level alpha=0.01

Supplementary Figure 2. Scatter plots and relative linear regressions of cell concentration of *P. multistriata* vs environmental parameters measured at LTER-MC at the sampling dates in which the species was recorded during the years 2008-2020: temperature (a), salinity (b), nitrate (c), and phosphate (d) concentrations. Spearman-based correlation matrix (e) calculated with the same data; PN = *P. multistriata* abundances, TEMP = temperature, PSAL = salinity, NTRA = nitrates, PHOS = phosphates.

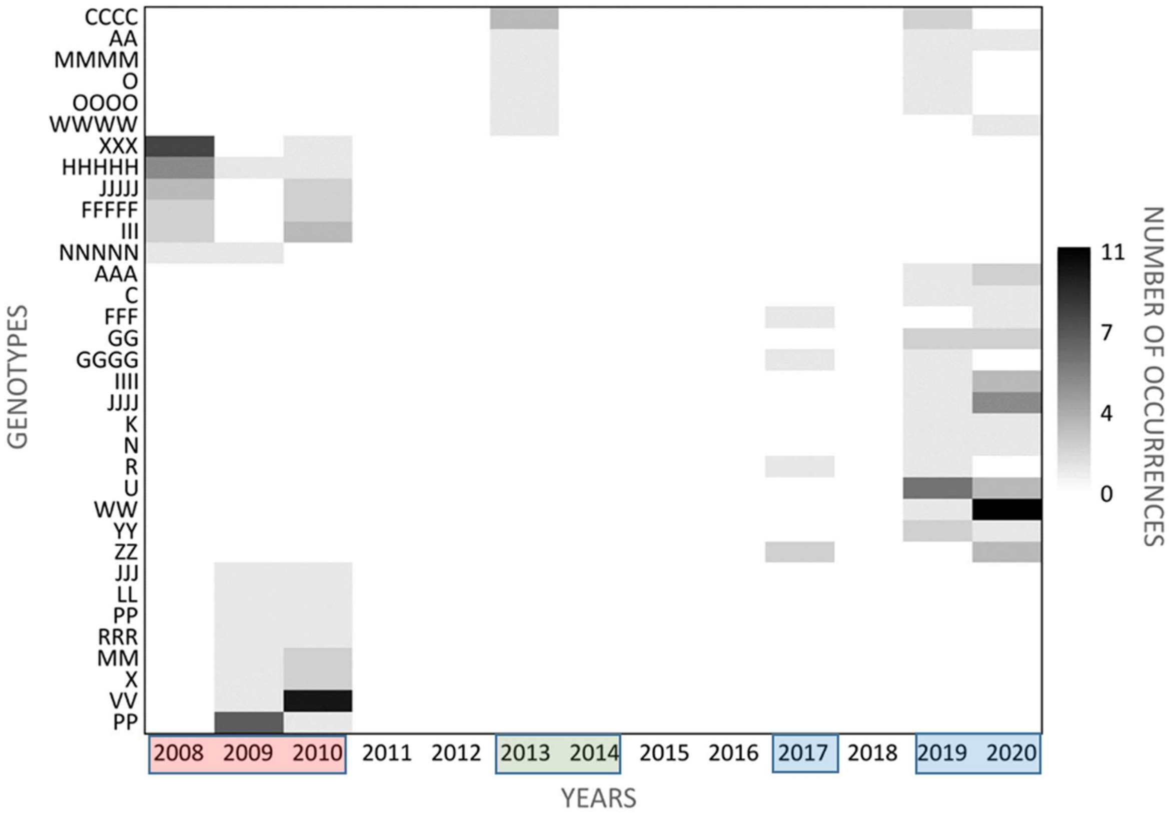

Supplementary Figure 3. Heat map illustrating the number of occurrences of the 34 genotypes that were resampled in different years. The years in which microsatellite fingerprinting was carried out are highlighted with the colour code used in Fig. 1.

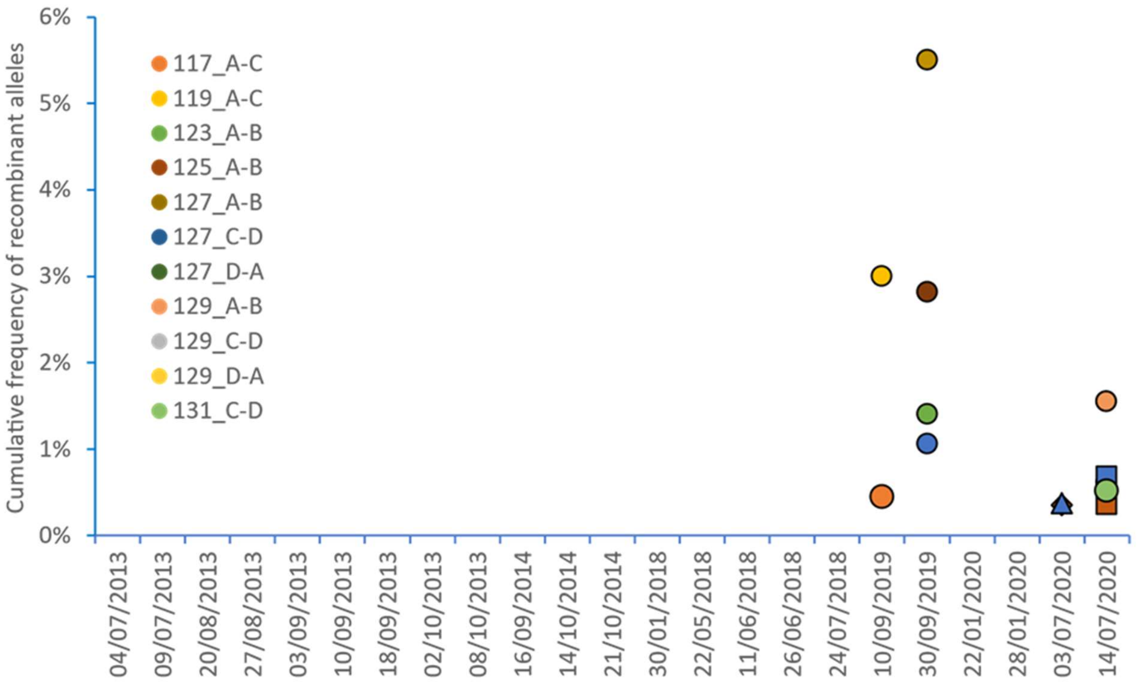

Supplementary Figure 4. Frequency, in percentage, of the recombinant alleles of locus PNm1 (see also Supplementary Table 4).

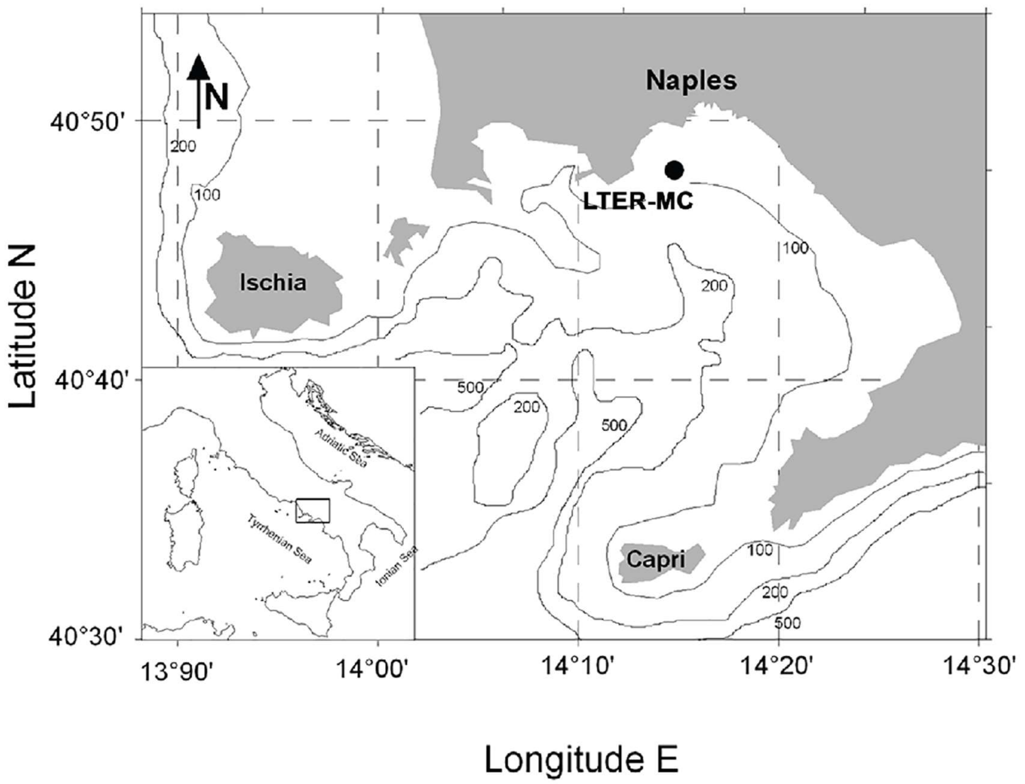

Supplementary Figure 5. Location of the Long Term Ecological Research site MareChiara (LTER-MC) in the Gulf of Naples Tyrrhenian Sea, Mediterranean Sea

SUPPLEMENTARY TABLES

Supplementary Table 1: Summary of the *Pseudo-nitzschia multistriata* datasets presented in this study. Seawater samples used to assess cell abundances, to isolate the strains genotyped with microsatellites and to extract DNA for Microsatellite Pool-seq Barcoding were collected at the LTER-MC station in the Gulf of Naples. The data on cell abundances for the period 1995-2000 are illustrated in Supplementary Figure 1 and those for the period targeted in this study (2008-2020) are illustrated in Fig. 1a. The data on microsatellite fingerprinting are illustrated in Fig. 1b, Fig. 2, Fig. 3. The data on Microsatellite Pool-seq Barcoding are illustrated in Fig. 4. Colour coding in the third column is the same as in Fig. 1.

**YEAR**	**Cell concentration**	**Microsatellite fingerprinting**	**Microsatellite Pool-seq Barcoding**
1995-2007	X		
2008	X	X	
2009	X	X	
2010	X	X	
2011	X		
2012	X		
2013	X	X	X
2014	X	X	X
2015	X		
2016	X		
2017	X	X	X
2018	X		X
2019	X	X	X
2020	X	X	X

Supplementary Table 2: Summary statistics for the samples of *Pseudo-nitzschia multistriata* collected in 2008–2020 and genotyped by fragment analysis. For each sampling date are reported: code of the LTER-MC sample, cell counts (cells L^{-1}), number of genotyped strains (N), number of multilocus genotypes (MLGs), genotypic richness (R_{MLG}), number of alleles (Na), number of strains for which MT was assessed (Strains MT), mating type ratio (MT ratio), index of association (I^A_S) and its statistical significance (* < 0.5; ** <0.01; *** <0.001). When cell concentration is zero, it means that *P. multistriata* cells were only recorded in net samples; nc = not calculated. For each year, the total number of isolated strains (N) and of strains for which MT was assessed (strains MT), are reported together with the average values (± st. dev.) for MLGs, $R_{MLG,}$ Na and MT ratio, and the value of I^A_S calculated considering all strains isolated in the year.

Year	Name	Sampling date	Cell counts	N	MLGs	R_{MLG}	Na	Strains MT	MT ratio	I^A_S
**2008**	MC 830	17 Sep 2008	0.00E+00	21	12	0.55	19	11	1	0.1275*
	MC 831	23 Sep 2008	1.32E+04	53	22	0.4	21	15	1	0.1987**
	MC 832	30 Sep 2008	0.00E+00	44	29	0.65	30	16	1	0.0409
	MC 834	14 Oct 2008	3.29E+03	18	9	0.47	15	11	1	-0.0315
	MC 837	04 Nov 2008	0.00E+00	7	5	0.67	12	Nc		0.329*
	MC 838	11 Nov 2008	9.88E+03	7	4	0.5	13	7	1	0.0505
	MC 839	18 Nov 2008	0.00E+00	7	5	0.67	17	7	1	0.6082**
				**157**	**12.29 (±9.66)**	**0.56 (±0.11)**	**18.14 (±6.12)**	**67**	**1.00 (0.00)**	**0.1369*** **
**2009**	MC 868	30 Jun 2009	1.11E+04	24	24	1	22	12	0.58	0.0005
	MC 869	7 Jul 2009	2.22E+04	35	30	0.85	19	27	0.44	0.0257
	MC 870	14 Jul 2009	0.00E+00	18	14	0.76	16	15	0.53	0.0852*
	MC 880	22 Sep 2009	0.00E+00	8	8	1	14	Nc		0.1045*
	MC 881	29 Sep 2009	0.00E+00	57	30	0.52	23	Nc		0.0783**
	MC 882	6 Oct 2009	3.29E+03	24	11	0.43	20	Nc		0.3486**
	MC 884	20 Oct 2009	2.64E+04	14	8	0.54	13	Nc		0.3104**
	MC 885	27 Oct 2009	0.00E+00	13	6	0.42	13	Nc		0.0964
				**193**	**16.38 (±10.08)**	**0.69 (±0.24)**	**17.50 (±4.04)**	**54**	**0.52 (0.07)**	**0.1217*** **
**2010**	MC 917	15 Jun 2010	4.43E+04	9	6	0.63	14	Nc		0.2114**
	MC 920	6 Jul 2010	4.65E+05	14	9	0.62	16	Nc		0.1617**
	MC 925	10 Aug 2010	3.77E+05	33	33	1	25	5	0.20	0.0248
	MC 929	7 Sep 2010	1.55E+05	48	44	0.91	28	16	0.38	0.0125
	MC 930	14 Sep 2010	6.59E+03	32	28	0.87	20	Nc		0.0022
	MC 931	21 Sep 2010	0.00E+00	26	17	0.64	16	12	0.58	0.3246**
				**162**	**22.83 (±14.74)**	**0.78 (±0.17)**	**19.83 (±5.60)**	**33**	**039 (0.19)**	**0.0163**
**2013**	MC1041	5 Feb 2013	1.32E+04	4	4	1	13	Nc		0.05
	MC1063	16 Jul 2013	3.29E+03	15	10	0.64	14	24	0.63	0.0313
	MC1065	6 Aug 2013	0.00E+00	4	4	1	11	44	0.8	0.0773
	MC1068	20 Aug 2013	1.58E+05	45	28	0.61	23	78	0.78	0.1781**
	MC1069	27 Aug 2013	3.77E+05	10	2	0.11	8	13	1	0.0057**
	MC1070	3 Sep 2013	3.21E+05	31	11	0.33	16	59	0.93	0.4898**
	MC1071	10 Sep 2013	4.96E+05	6	3	0.4	9	15	1	0.5536**
	MC1072	18 Sep 2013	2.76E+04	25	10	0.38	16	31	0.9	0.2837**
	MC1073	24 Sep 2013	2.32E+04	38	3	0.05	10	51	0.94	0.3936**
	MC1074	2 Oct 2013	2.63E+05	61	4	0.05	9	65	0.97	-0.0055

	MC1075	8 Oct 2013	1.33E+05	38	3	0.05	13	57	1	0.8092**
	MC1076	15 Oct 2013	3.29E+04	28	4	0.11	10	31	1	0.6635**
	MC1077	22 Oct 2013	0.00E+00	33	2	0.03	10	33	0.97	0.5014**
	MC1078	28 Oct 2013	0.00E+00	26	3	0.08	10	26	1	0.1546**
	MC1079	07 Nov 2013	0.00E+00	26	1	0	7	26	0.96	0.8092**
				**390**	**6.13 (±6.81)**	**0.32 (±0.35)**	**11.93 (±4.10)**	**553**	**0.92 (0.11)**	**0.4684*** **
**2014**	MC1112	29 Jul 2014	0.00E+00	3	3	1	10	Nc		Nc
	MC1119	2 Sep 2014	0.00E+00	2	2	1	11	Nc		Nc
	MC1120	9 Sep 2014	1.32E+04	8	8	1	17	13	0.38	0.1675**
	MC1124	7 Oct 2014	1.11E+04	7	7	1	16	8	0.63	0.1099
	MC1125	14 Oct 2014	4.43E+04	2	2	1	10	Nc		Nc
	MC1126	21 Oct 2014	4.65E+04	2	2	1	11	Nc		Nc
				**24)**	**4 (±2.76)**	**1 (±0.00)**	**12.50 (±3.15)**	**21**	**0.50 (0.17)**	**0.0228**
**2017**	MC1264	29 Aug 2017	1.65E+04	14	12	0.85	15	16	0.81	-0.0099
	MC1266	12 Sep 2017	0.00E+00	8	7	0.86	13	6	0.33	-0.0057
				**22**	**19**	**0.86**	**15**	**22**	**0.57**	**0.0067**
**2019**	MC1334	21 Jun 2019	0.00E+00	6	5	0.8	11	8	0.63	0.0268
	MC1336	7 Jul 2019	0.00E+00	2	2	1	11	Nc		nc
	MC1339	1 Aug 2019	0.00E+00	9	9	1	12	Nc		-0.0437
	MC1340	5 Aug 2019	0.00E+00	6	5	0.8	13	15	0.27	0.4029**
	MC1342	3 Sep 2019	1.39E+05	28	25	0.89	17	29	0.45	0
	MC1343	10 Sep 2019	4.43E+05	8	8	1	17	Nc		0.1642*
	MC1344	17 Sep 2019	3.29E+03	15	12	0.79	18	22	0.59	0.0106
	MC1345	24 Sep 2019	0.00E+00	1	1	1	7	nc		nc
	MC1346	30 Sep 2019	7.75E+04	3	3	1	12	nc		nc
				**78**	**7.78 (±7.36)**	**0.92 (±0.10)**	**13.11 (±3.59)**	**74**	**0.48 (0.13)**	**0.0156**
**2020**	MC1355	14 Jan 2020	0.00E+00	19	17	0.89	14	51	0.47	-0.0022
	MC1356	22 Jan 2020	2.77E+04	33	26	0.78	15	30	0.5	0.0284
	MC1357	28 Jan 2020	1.71E+04	20	19	0.95	16	20	0.6	0.0207
	MC1359	24 Feb 2020	3.29E+03	6	6	1	16	15	0.47	-0.0571
	MC1364	7 Jul 2020	0.00E+00	11	7	0.6	14	nc		0.2391**
	MC1365	14 Jul 2020	2.22E+04	18	10	0.53	13	29	0.55	0.3845**
				**107**	**14.17 (±7.83)**	**0.79 (±0.19)**	**14.67 (±1.21)**	**145**	**0.52 (0.06)**	**0.0786*** **

Supplementary Table 3: Summary statistics for the environmental samples collected in 2013–2020 and genotyped via Microsatellite Poolseq Baracoding (MPB). For each sampling date are reported: code of the LTER-MC sample, the number of reads (N reads), the total number of alleles (Na) and the number of homoplastic alleles (Noa).

Year	MC code	Sampling date	N reads	Na	Noa
2013	MC 1061	04 Jul 2013	106 326	23	17
	MC 1062	09 Jul 2013	111 187	23	16
	MC 1068	20 Aug 2013	106 486	20	14
	MC 1069	27 Aug 2013	108 274	21	15
	MC 1070	3 Sep 2013	109 028	19	14
	MC 1071	10 Sep 2013	110 416	18	13
	MC 1072	18 Sep 2013	111 371	18	12
	MC 1074	2 Oct 2013	111 043	21	14
	MC 1075	8 Oct 2013	111 233	19	12
		**2013**	**109 485 (±2045)**	**20.22 (±1.92)**	**14.11 (±1.69)**
2014	MC 1121	16 Sep 2014	106 878	27	20
	MC 1125	14 Oct 2014	109 251	17	14
	MC 1126	21 Oct 2014	108 966	25	19
		**2014**	**108 365 (±1296)**	**23.00 (±5.29)**	**17.67 (±3.21)**
2018	MC 1280	30 Jan 2018	109 466	25	18
	MC 1294	22 May 2018	110 761	25	16
	MC 1297	11 Jun 2018	106 284	23	18
	MC 1299	26 Jun 2018	110 373	18	14
	MC 1302	24 Jul 2018	111 663	21	17
		**2018**	**109 709 (±2071)**	**22.40 (±2.97)**	**16.60 (±1.67)**
2019	MC1343	10 Sep 2019	111 019	27	20
	MC1346	30 Sep 2019	110 899	23	16
		**2019**	**110,959 (±85)**	**25.00 (±2.83)**	**18.00 (±2.83)**
2020	MC1356	22 Jan 2020	109 290	25	19
	MC1357	28 Jan 2020	109 143	25	18
	MC1363	3 Jul 2020	108 697	36	25
	MC1365	14 Jul 2020	107 193	43	31
		**2020**	**108 580 (±959)**	**32.25 (±8.85)**	**23.25 (±6.02)**

Supplementary Table 4: List of PNm1 alleles and their composition. Each letter represents a different flanking sequence. Colours indicate the different combinations of 5' and 3' blocks. Small caps letters in the "Repeat" column indicate different SNPs in the repeat regions;"+1nt 5'" indicates the presence of one extra-nucleotide at the 5' end.

Allele	5'	Repeat	3'
115_14	A	(TC)6	A
117_7	A	(TC)7	A
119_3	A	(TC)8	A
119_11	A	(TC)8a	A
119_16	A	(TC)8b	A
121_9	A	(TC)9	A
121_1	A	(TC)9a	A
123_6	A	(TC)10	A
125_5	A	(TC)11	A
125_17	A	(TC)11a	A
127_12	A	(TC)12a	A
127_4	A	(TC)12	A
129_2	A	(TC)13	A
129_27	A	(TC)13a	A
129_1000	A	(TC)13b	A
131_8	A	(TC)14	A
133_13	A	(TC)15	A
145_21	A	(TC)20	A
147_22	A	(TC)22	A
123_51	A	(TC)10	B
125_3	A	(TC)11	B
127_19	A	(TC)12	B
129_108	A	(TC)13	B
117_97	A	(TC)7	C
119_29	A	(TC)8	C
122_44	B	(TC)9 +1nt 5'	A
124_2	B	(TC)10+1nt 5'	A
126_15	B	(TC)11+1nt 5'	A
128_26	B	(TC)12 +1nt 5'	A
127_63	C	(TC)12	D
129_45	C	(TC)13	D
131_53	C	(TC)14	D
127_57	D	(TC)12	A
129_5	D	(TC)13	A

SUPPLEMENTARY METHODS

SM 1: Microsatellite PoolSeq Barcoding analysis

Environmental DNA was extracted from the 23 field samples using NucleoSpin® Soil kit (Macherey-Nagel GmbH, Duren, Germany). In the first PCR, three replicates per environmental DNA sample were run for each locus using 5 ng/µL of eDNA (15 ng/µL for samples whose PCR product was not detected on 1.5 % agarose gel), 2x KAPA HiFi HotStart ReadyMix, and the Illumina primers for locus PNm1(F: CACCAATTGCATCCTAAAAGGG and R: CCGTCTAAGCCTGTATTTGTGAC) and locus PNm6420 (F:GAAGCCTCCTATTGCTGCAT and R:ACTGCATTCCAGGATTGGTC). The PCR was performed in a thermal cycler (Mastercycler Nexus gradient, Germany) under the following conditions: 5 min at 94°C prior to 40 cycles of 30 s at 94°C, 90 s at 59°C, 40 s at 72°C and a final elongation step at 72°C for 10 min for PNm1 and PNm6420. Triplicate PCR products were pooled and visualised on a 1.5% agarose gel. PNm1 and PNm6420 bands were cut around 100- 250 bp and 100-300 bp, respectively, and purified using NucleoSpin Gel and PCR Clean-up Kit (Macherey-Nagel, Düren, Germany). A second PCR reaction was performed to attach the dual indices and Illumina sequencing adapters to the first PCR products using the Nextera XT Index Kit. The final PCR products were purified using NGS clean-up mag-beads (Beckman Coulter, Brea, CA, USA), validated using LabChip GX Touch Nucleic Acid Analyzer (Agilent Technologies, Santa Clara, CA, USA) and pooled at equimolar ratios. MPB libraries were sequenced using the MiSeq System with the v3 2 × 300 bp Paired-end Kit (Illumina).

SM 2: Correction for PCR artefacts

a) Preferential amplification in PNm1 and PNm6420 microsatellites

Preferential amplification is the differential amplification of PCR products representing two alleles in a heterozygous sample. This phenomenon can result in imbalanced amplification favouring the shorter allele over to the longer one. The extent of preferential amplification is usually directly proportional to the size difference between the allelic PCR products, and it increases with the number of cycles of the PCR (Walsh et al., 1992).

Preferential amplification is excluded in the allele frequency tables produced by fragment analysis but is included in those obtained from MPB. To account for preferential amplification, the peak height of 82 (locus PNm1) and 67 (locus PNm6420) genotypic profiles of heterozygous strains genotyped through capillary electrophoresis was considered. The peak height ratio for each heterozygous strain (i.e., 100%) was determined in RFUs. The median of heterozygous peak height ratio for PNm6420 (28.9%) and PNm1 (68.5%) indicates that preferential amplification occurred, especially for PNm6420 that spans a wider range of allele sizes (Figure SM1).

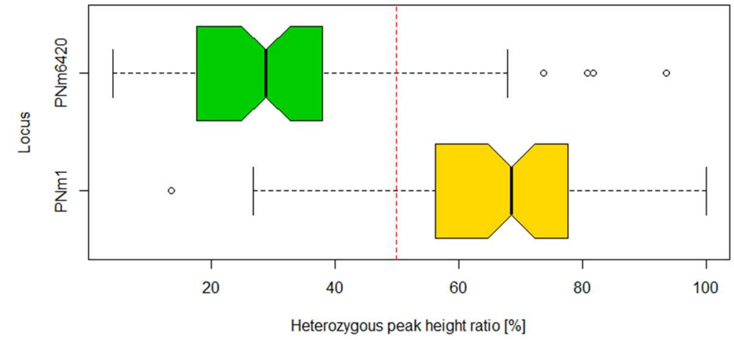

Figure SM1: Preferential amplification by locus. Distribution of heterozygous peak height ratio by locus for n=82 and n=67 heterozygous strains, respectively. The vertical red line is at 50%. The black central mark in the notch indicates the median, the bottom and top edges of the notch are the 25th and 75th percentile. The whiskers extend to the most extreme data points not considered outliers, and the outliers are plotted individually as white dots.

To correct for preferential amplification in the artificial DNA mixtures for Validation 1, we used the electropherograms of 5 strains of *P. multistriata* (Table SM1). The corrected contribution of each allele in the

mix was determined based on the theoretical contribution of the allele in the mix and the corresponding peak heights in the single strain analysis: each allele frequency in the mix was calculated by multiplying the theoretical allele contribution (based on the genotype DNA added) with the relative peak height of each allele in the single strain analysis (Equation1)

$$Allele\ frequency\ (Mix) = Theoretical\ allele\ contribution\ (Mix) \cdot \frac{Allele\ peak\ (Strain)}{Sum\ allele\ peaks\ (Strain)} \quad (1)$$

Correction for preferential amplification was only possible in Validation 1, because electropherograms were not available for all sampling dates.

Supplementary Methods Table 1 (Table SM1): Strains selected for artificial DNA mixtures and correspondent genotypes for the PNm1 and PNm6420 loci.

Strain code	PNm1		PNm6420	
MC 1339_8	127	127	125	164
MC 1339_11	119	119	112	164
MC 1342_35	117	127	112	164
MC 1343_46	119	127	164	164
MC 1343_47	117	119	112	164

b) Stutter products in in PNm1 and PNm6420 microsatellites

The second source of error concerning microsatellite amplification is stutter banding. When diverse samples including many individuals are studied, these bands can overlap with the correct-sized alleles.

Stutters were detected for both microsatellite loci in electropherograms as well as MPB sequences but are usually excluded in fragment analysis while they are included in MPB frequency tables. To compare the allele frequency tables produced by the two methods, theoretical stutters were added to fragment analysis allele frequency tables in Validation 1 and 2. Allele frequencies were corrected for stutter presence using stutter correction factors, determined according to equation 2.

$$Stutter\ correction\ factor = 100\ x\ \frac{Stutter\ peak\ height\ (RFUs)}{Parent\ allele\ peak\ height\ (RFUs)} \quad (2)$$

Stutter correction factors of the stutters associated with each allele were calculated as an average of 95 strains genotyped through capillary electrophoresis (Table SM2). The values obtained (stutter correction factors) were used to estimate stutter frequency according to equation 3.

.

$$Stutter\ f = \ Stutter\ correction\ factor\ x\ Parent\ allele\ f \quad (3)$$

Stutter frequencies were added to the final allele frequency tables. Parent alleles and stutters frequencies were then adjusted to a total sum of 100%.

Supplementary Methods Table 2 (Table SM2): Mean stutter percentage at PNm1 and PNm6420 loci calculated over 95 strains genotyped through capillary electrophoresis.

PNm1		
**Parent allele**	**Stutter**	**Mean stutter percentage (%)**
119	115	3,01

	117	10,23
121	115	3,38
	117	6,46
127	119	3,21
	123	8,14
129	125	19,42
	123	2,07
	125	3,44
	127	18,6
	131	4,13
147	143	12,64
	145	37,54
149	143	5,82
	145	17,28
	147	45,73
	151	9,49

PNm6420		
**Allele**	**Stutter**	**Mean stutter percentage (%)**
114	115	10,8
126	123	5,82
129	123	1,3
	126	8,76
138	134	2,35
	136	13,97
147	141	2,88
	144	17,36
165	156	1,78
	159	6,27
	162	26,69

SM 3: Validation of MPB results by comparison to fragment analysis

a) Validation 1 (Artificial mixtures)

In Validation 1 allele frequency tables obtained by MPB method were compared to theoretical allele frequency tables of artificial mixtures of DNA for fragment analysis. These theoretical allele frequency tables were constructed for each locus based on electropherograms of the 5 strains included in the artificial mixtures of DNA. They were corrected for preferential amplification of heterozygous genotypes as well as for stutter presence (see SM2). Finally, the corrected single strain allele frequencies were combined according to the applied proportions in the final artificial mixtures of strain DNA (Table SM3). Since fragment analysis only provides information on allele length, all the homoplastic alleles in MPB allele frequency tables were summed up for validation. Allele frequencies (%) produced by the two methods were compared for each sample by linear regression (Pearson's r coefficient, Figure SM2) and their mean deviation (i.e., error) was determined (Table SM4). Principal component analysis (PCAs) of the two datasets was performed and visualised in ordination plots (Figure SM3).

Supplementary Methods Table 3 (Table SM3): Artificial DNA mixtures composition, genotypes employed and proportions

	MC 1339_8	MC 1339_11	MC 1342_35	MC 1343_46	MC 1343_47
**Mix 1**	5 ng/µL				
**Mix 2**	5 ng/µL	5 ng/µL			
**Mix 3**	5 ng/µL		5 ng/µL		
**Mix 4**	5 ng/µL			5 ng/µL	
**Mix 5**	5 ng/µL				5 ng/µL
**Mix 6**		5 ng/µL			
**Mix 7**		5 ng/µL	5 ng/µL		
**Mix 8**		5 ng/µL		5 ng/µL	
**Mix 9**		5 ng/µL			5 ng/µL
**Mix 10**			5 ng/µL		
**Mix 11**			5 ng/µL	5 ng/µL	
**Mix 12**			5 ng/µL		5 ng/µL
**Mix 13**				5 ng/µL	
**Mix 14**				5 ng/µL	5 ng/µL
**Mix 15**					5 ng/µL
**Mix 16**	0,1 ng/µL	1 ng/µL	10 ng/µL		
**Mix 17**	10 ng/µL	0,1 ng/µL	1 ng/µL		
**Mix 18**	1 ng/µL	10 ng/µL	0.1 ng/µL		
**Mix 19**	2,5 ng/µL	0,25 ng/µL	1,25 ng/µL	0,5 ng/µL	0,5 ng/µL

The correlations between both methods in Validation 1 was high and significant for both microsatellite loci (Pearson's $r = 0.91$, $p < 0.001$, see Figure SM2). The mean allele frequency error was 3.87 ± 4.25 % for alleles of PNm1 and 5.07 ± 3.66 % for PNm6420. These results point out that allele identification and quantification were similarly accurate in both methods, bearing in mind all the previous premises. The PCA plots support visually the similarity of samples analysed through both methods (Figure SM3).
Since alleles with the highest mean error are those with high mean stutter percentage (Table SM4), the detected differences might be related to inconsistencies in stutter behaviour, which was merely calculated theoretically for the fragment analysis dataset.

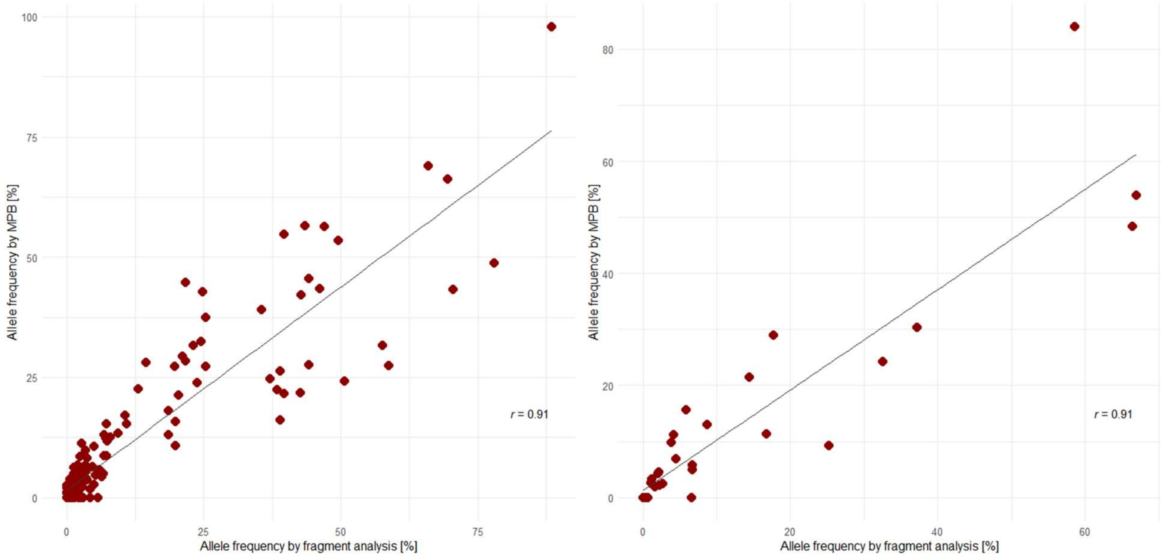

Figure SM2: Validation step 1 results of relative allele abundances theoretically constructed from fragment analysis data (x-axis) and produced by MPB (y-axis) using PNm1 (a) and PNm6420 (b) primer with artificial DNA mixtures of 5 strains, i.e., with limited diversity. Pearson's correlation coefficient (r) was used to evaluate the linear regression.

Supplementary Methods Table 4 (Table SM4): Mean relative abundance of alleles at PNm1 using MPB (%) and fragment analysis(%) for artificial DNA mixtures, and mean error rate between the two methods (%). Alleles that showed a high mean stutter percentage are indicated with *.

PNm1			
**Allele**	**Mean allele abundance Fragment Analysis (%)**	**Mean allele abundance MPB (%)**	**Mean error (%)**
X115	1.59	NA	1.59
X117	3.68	2.41	1.79
X119	15.43	17.42	2.66
X121*	34.22	40.23	8.21
X123	0.73	1.41	0.74
X125	1.21	4.36	3.16
X127*	6.54	11.19	4.65
X129*	35.14	20.41	14.73
X131	1.45	2.51	1.08
X133	NA	0.05	0.05
**PNm6420**			
**Allele**	**Mean allele abundance Fragment Analysis (%)**	**Mean allele abundance MPB (%)**	**Mean error (%)**
X114*	19.88	30.27	10.68
X115	3.07	1.18	1.90
X123	1.62	3.58	2.17
X126*	27.86	22.52	5.34
X156	0.63	0.83	0.86
X159	2.21	4.93	2.72
X162	9.41	13.42	4.93
X165*	35.24	23.27	11.97

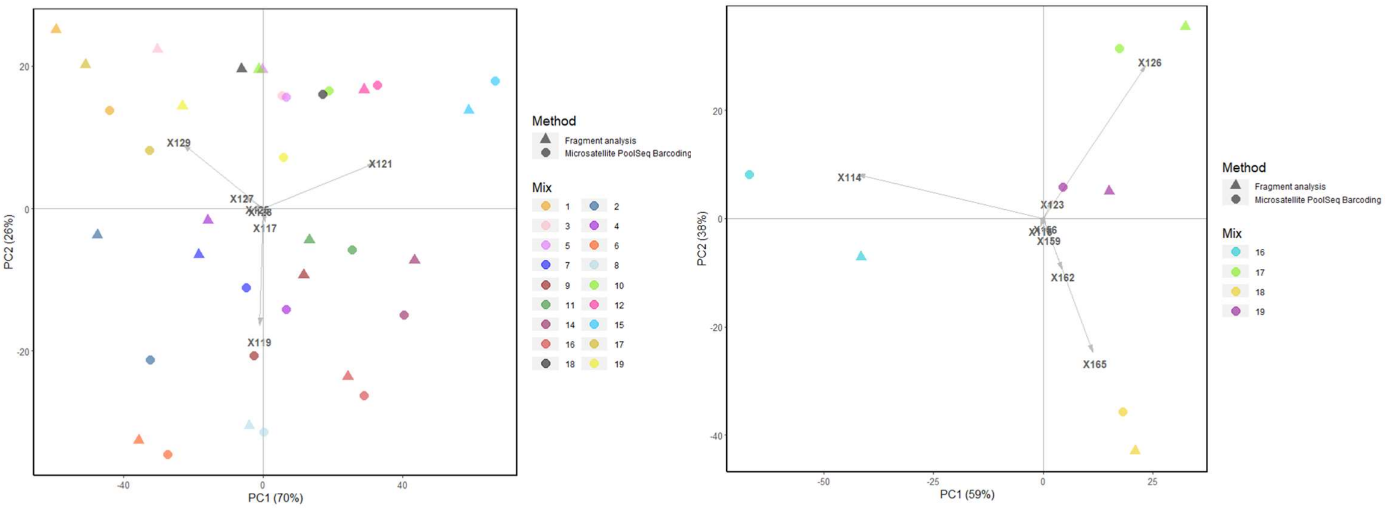

Figure SM3: Validation 1 (MPB vs traditional method) of PNm1 and PNm6420. PCA of allele frequencies in artificial DNA mixtures in Validation 1 as measured by MPB and fragment analysis of microsatellite locus PNm1. Each dot represents a mix and the arrows pointed out each variable, allele. The method by which the result was obtained is marked by different shapes.

b) Validation 2 (Environmental samples)

In the second validation step, allele frequency tables produced by MPB analysis on environmental samples collected during 2013, 2014 and 2020 bloom events were compared with those theoretically constructed from 666 genotypes of *P. multistriata* individuals collected at the same time points. Theoretical allele frequency tables for fragment analysis were obtained for each sampling date by calculating the relative proportion of each allele for that time point from the available genotypes. The allele frequencies obtained were corrected for stutter presence by using stutter correction factors (see SM2b). As in Validation 1, homoplastic alleles in the MPB dataset were summed up and allele frequencies were compared for each sample by linear regression (Pearson's r coefficient, Figure SM4) and their mean deviation (i.e., error) was determined (Table SM5). The correlation coefficient was calculated for the years under investigation considered as a whole, as well as for each bloom event separately (Summer 2013, summer 2014, winter 2020, summer 2020). Principal component analysis (PCAs) of the two datasets was performed and visualised in ordination plots (Figures SM5 and SM6). In Validation 2 the MPB method showed a larger deviation from allele frequencies theoretically constructed from fragment analysis data and displayed a higher variability among the two loci (Figure SM4). Pearson's r coefficient was significant ($p < 0.001$) for both loci with values equal to $r = 0.71$ for PNm1 and $r = 0.47$ for PNm6420. The mean frequency error per allele was 4.14 ± 6.60 % for PNm1 and 4.88 ± 8.90% for PNm6420.

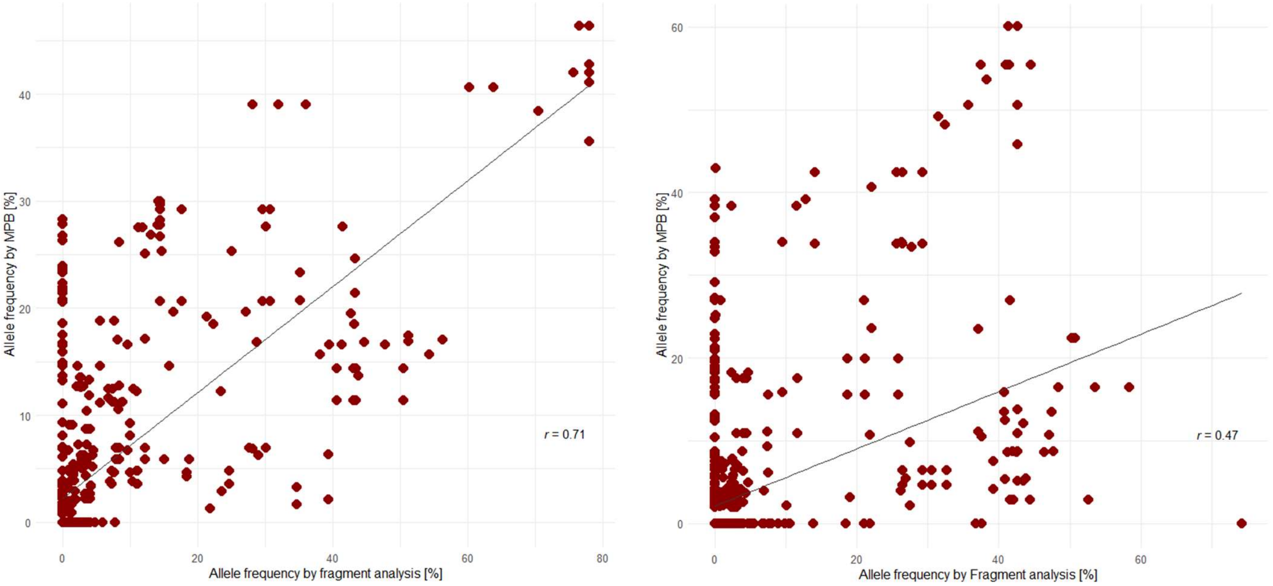

Figure SM4: PNm1 and PNm6420 Validation 2 (MPB vs traditional method). Results of validation 2 of relative allele frequencies for each allele as measured by fragment analysis (x-axis) and by MPB (y-axis) using PNm1 (a) and PNm6420 (b) primers for natural community samples. Genotype analyses were based on a total of 639 (a) and 623 (b) strains covering different blooms that occurred during 2013, 2014 and 2020.

Supplementary Methods Table 5 (Table SM5): Mean relative abundance of alleles at PNm1 using MPB (%) and fragment analysis (%) for natural community samples, and mean error rate between the two methods (%). Alleles that showed a high mean stutter percentage are indicated with *.

PNm1			
Allele	Mean allele abundance Fragment Analysis (%)	Mean allele abundance MPB (%)	Mean error (%)
X115	1.11	0.12	1.05
X117 *	10.31	3.34	8.60
X119	12.57	15.10	6.11
X121 *	2.42	12.95	13.08
X122	NA	0.03	NA
X123	2.42	4.41	2.52
X124	NA	0.09	0.09
X125 *	5.91	10.80	6.61
X126	NA	0.22	0.22
X127 *	27.37	20.87	19.74
X128	NA	0.04	0.00
X129 *	34.49	27.90	24.26
X131	1.42	3.87	2.45
X133	NA	0.20	0.20
X139	0.03	NA	0.03
X143	0.16	NA	0.16
X145	0.47	0.02	0.49
X147	1.26	0.04	1.30
X149	0.02	NA	0.02
X151	0.00	NA	0.00
X195	0.02	NA	0.02

PNm6420			
Allele	Mean allele abundance Fragment Analysis (%)	Mean allele abundance MPB (%)	Mean error (%)
X114*	13.34	16.80	13.43
X115	1.44	1.81	1.85
X117	0.25	0.26	0.51
X120	NA	0.22	0.22
X123	0.27	2.05	1.90
X126*	4.02	13.25	10.04
X128	1.59	NA	1.59
X129*	3.98	16.18	14.51
X131	0.18	NA	0.18
X132*	14.82	28.31	14.70
X134	0.28	NA	0.28
X135	0.50	0.69	0.35
X138	0.31	NA	0.31
X141	0.01	NA	0.01
X144	0.04	0.19	0.24
X147	0.25	0.39	0.64
X150	0.02	0.05	0.06
X153	NA	0.17	0.17
X156	0.84	0.54	1.10
X159	2.69	3.35	1.69
X162*	11.48	7.17	32.66
X163	0.07	NA	0.07
X165*	41.19	8.52	32.66

X168	1.75	0.06	1.80
X179	0.63	NA	0.63
X242	0.03	NA	0.03
X245	0.05	NA	0.05

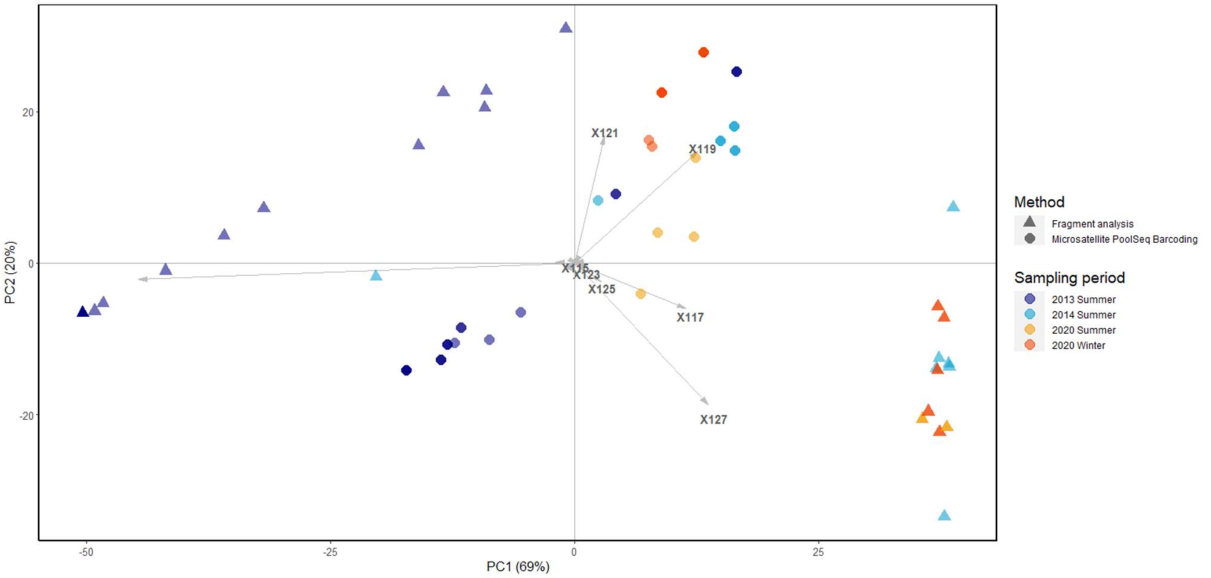

Figure SM5: PNm1 Validation 2 (MPB vs traditional method). PCA of allele frequencies in natural community samples in Validation 2 as measured by MPB and fragment analysis of microsatellite locus PNm1. Each dot represents a natural sample, and the arrows point out each variable, allele. The method by which the result was obtained is marked by different shapes.

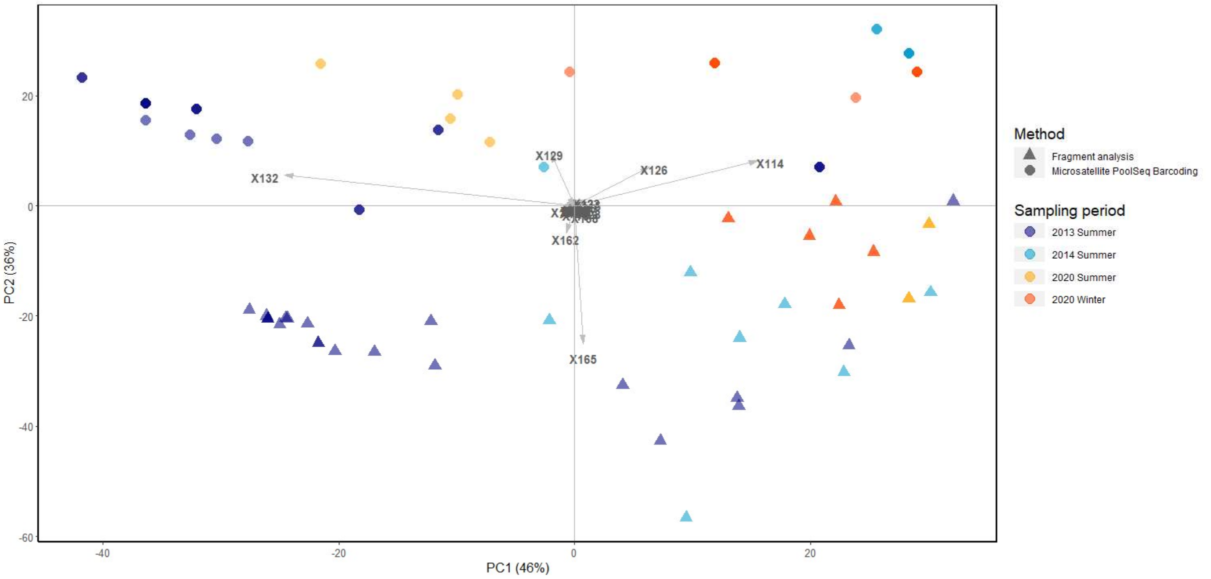

Figure SM6: PNm6420 Validation 2 (MPB vs traditional method). PCA of allele frequencies in natural community samples in Validation 2 as measured by MPB and fragment analysis of microsatellite locus PNm1. Each dot represents a natural sample, and the arrows point out each variable, allele. The method by which the result was obtained is marked by different shapes.

The low correlation coefficients detected in the environmental samples in Validation 2 can be attributed to several factors, including statistical, ecological, and technical aspects, such as differences in sample size, genetic diversity of a bloom, and the exclusion of preferential amplification in the fragment analysis correction of Validation 2.

A significant factor is the limited sample size used in traditional genotyping, which involves isolating single cells of *P. multistriata*. Thus, it represents only a very small sub-sample (max. a few hundred cells) of the species' actual population (which typically ranges from 10^4-10^5 cells L^{-1} during bloom events, see Figure 1a). This subsampling bias is much smaller in the MPB method, which resolves alleles of all individuals present in the sampled volume of water (e.g., all cells in 1L) at a specific time and place, making the sample more representative and increasing the statistical power. In Figure SM4, for example, the MPB method detected numerous alleles that were missed by fragment analysis, indicated by the dots on the y-axis. Furthermore, abundant alleles may appear inflated at the expense of rare ones due to their relative abundance in a restricted sample size.

To assess the effect of sample size on the correspondence between the two methods, the comparison between allele frequency (%) tables at PNm1 and PNm6420 was conducted separately for each bloom event (Summer 2013, summer 2014, winter 2020 and summer 2020), where genotyping was based on different sample sizes of isolated genotypes. Results highlighted that the two methods correlated much better during time periods in which a high number of strains was isolated and genotyped (Table SM6). Highest Pearson's R values for both loci correspond to the summer bloom of 2013 during which a high number of strains (481) was genotyped. The locus PNm6420 also showed high r values during the winter bloom of 2020, where 99 strains were isolated. Accordingly, the goodness of fit (indicated by Pearson's r coefficient) and the number of strains isolated for each period measured at both loci were positively correlated. It is important to note, however, that summer 2013 samples correspond to the period when the *P. multistriata* population experienced a clonal expansion (Ruggiero et al., 2018). The fact that the fit is better when natural diversity is low and/or isolated strain number is high points towards the statistical drawbacks of single-strain genotyping being the main source of discrepancy between the methods. However, the clusters detected over time at both loci are quite robust and consistent between the two methods (Figure SM5 and SM6).

Supplementary Methods Table 6 (Table SM6): Number of strains, Pearson's r coefficient and p-value for each sampling period for PNm1 and PNm6420 loci.

		**PNm1**	
**Sampling period**	**Number of strains genotyped**	**Pearson's r coefficient- MPB vs Fragment Analysis**	**p-value**
Summer 2013	481	0.87	< 0.001
Summer 2014	34	0.50	< 0.001
Winter 2020	99	0.42	< 0.001
Summer 2020	29	0.44	< 0.001
		**PNm6420**	
**Sampling period**	**Number of strains genotyped**	**Pearson's r coefficient - MPB vs Fragment Analysis**	**p-value**
Summer 2013	468	0.63	< 0.001
Summer 2014	34	0.18	< 0.01
Winter 2020	97	0.60	< 0.001
Summer 2020	28	0.23	< 0.05

References

1. Walsh PS, Erlich HA, Higuchi R. Preferential PCR amplification of alleles: mechanisms and solutions. PCR Methods Appl. 1992 May;1(4):241-50. doi: 10.1101/gr.1.4.241. PMID: 1477658.

2. Ruggiero MV, D'Alelio D, Ferrante MI, Santoro M, Vitale L, Procaccini G, et al. Clonal expansion behind a marine diatom bloom. ISME J. 2018;12:463–72.
